# From public innovation procurement requirements to AI-enabled socio-technical architecture: a longitudinal case study in industrialized housing

**DOI:** 10.3389/frai.2026.1962132

**Published:** 2026-09-29

**Authors:** Alberto Cerezo-Narváez, MCarmen Guerrero Delgado, Magdalena Ramírez-Peña, Daniel Sánchez-García, José Sánchez Ramos

**Affiliations:** 1 Department of Mechanical Engineering and Industrial Design, Project Management and Engineering Research Group, University of Cadiz, Puerto Real, Spain; 2 Department of Energy Engineering, Termotecnia Research Group, University of Seville, Seville, Spain

**Keywords:** affordable housing, artificial intelligence, decision support systems, industrial transformation, industrialized housing, low-carbon construction, public innovation procurement, socio-technical systems

## Abstract

Artificial intelligence (AI) is used in digital systems that combine data, computational methods, decision processes, and organizational arrangements. This paper examines how the requirements and institutional conditions of a public innovation procurement process are reflected in the development and specification of an AI-enabled socio-technical architecture for industrialized public housing delivery. The case concerns an AI-enabled system intended to support the design, assessment, and procurement preparation of industrialized housing within a public-sector delivery process. Using a longitudinal qualitative pre-deployment case-study design, we analyze a documentary corpus covering institutional procurement records, the initial technical proposal, its formal ex-ante assessment, and subsequent collaborative development. The architecture documented in the case comprises eight interconnected functional modules and three cross-cutting enablers: security, governance, and interoperability. It combines rule-based regulatory and contextual checking, generative and parametric design configuration, surrogate-model-supported performance estimation, cost and financial assessment, multi-objective optimization, multicriteria scenario comparison, and procurement-document generation. Across the corpus, we identify traceable correspondences between institutional requirements and functional, computational, governance, and interoperability arrangements, while keeping requirements, specifications, formal ex-ante assessment, subsequent development, and evidence requiring operational validation distinct. The contribution is an account of how AI-related and other computational capabilities are integrated with data, human decision points, governance arrangements, and procurement outputs within an evolving socio-technical architecture. We do not evaluate a completed system or establish operational effectiveness, scalability, or broader public outcomes. It provides a pre-deployment account of how procurement and institutional requirements correspond to the architecture of an AI-enabled decision-support system for industrialized housing delivery.

## 1 Introduction

Artificial intelligence (AI) is increasingly embedded in the digital infrastructure used to plan, design, construct, and operate buildings. In the architecture, engineering, and construction (AEC) sector, current applications include automated rule checking and performance prediction. Other applications include design exploration, scheduling, and lifecycle-data analysis (Baduge et al., 2022; Gill et al., 2024). When combined with Building Information Modeling (BIM) and lifecycle assessment (LCA), AI can support decisions on materials, buildability, energy performance, and sustainability (Li et al., 2024). These applications are part of the Construction 4.0 transition, in which data and digital platforms are used to coordinate production and improve productivity (Balasubramanian et al., 2024). This matters particularly in construction, where project-based organization and discontinuous information flows still hinder coordination across design, manufacturing, logistics, and assembly. Reviews of off-site construction and BIM-enabled design for manufacturing and assembly (DfMA) consistently identify fragmented information and weak cross-stage integration as barriers to collaboration and production planning (Bakhshi et al., 2022; Cheng et al., 2023).

High initial costs and dependence on proprietary platforms can hinder AI adoption. Uneven data quality and algorithmic bias create additional risks. For public-sector use, governance, interoperability, and data quality become part of the technical problem rather than external considerations (Omrany et al., 2023; Wang et al., 2024). In Spain, public housing provides a particularly demanding policy and delivery context. Public authorities must reconcile affordability and access with regulatory compliance, while also addressing environmental performance, transparency, and the long-term stewardship of the public housing stock (Government of Spain, 2023; Ministry of Housing and Urban Agenda, 2023). Modular and prefabricated methods are often proposed as a response to housing demand because they can shorten construction programmes and reduce waste (Khan et al., 2023; Zohourian et al., 2025). Their effective use requires coordination from design and manufacturing through transport, assembly, and operation. BIM, IoT sensing, and lifecycle data platforms increasingly support this continuity (Parracho et al., 2025). AI-enabled digital twins may add real-time monitoring, predictive maintenance, and dynamic scheduling in off-site construction (Najafzadeh and Yeganeh, 2025), while lifecycle integration can support more sustainable building systems (Zhang Z. et al., 2024). Yet construction-AI research has mainly examined application-specific capabilities, digital integration, and performance-oriented use cases. Research on AI in public-sector settings has addressed a different set of questions, including organizational change, governance, accountability, risk, and procurement practices (Hickok, 2024; Johnson et al., 2025; Tangi et al., 2025). Innovation-procurement research has also examined the translation of public needs into functional requirements and the way problem and solution framings can evolve during procurement (Edquist and Zabala-Iturriagagoitia, 2020; Clarke et al., 2026). The computational techniques relevant to the present case, including rule-based checking, generative and parametric design, surrogate modelling, and multi-objective optimization, are established approaches in their respective research streams. The unresolved issue addressed here lies instead in the connection between institutional procurement requirements and the architecture in which such capabilities are organized for public-sector decision support. Less attention has been paid to how requirements concerning compliance, industrialization, performance assessment, interoperability, governance, and procurement readiness are reflected in the functional and computational configuration of such a system before operational deployment. Industrialized housing provides a particularly relevant setting because these technical capabilities must be connected to public accountability, affordability and environmental objectives, and the preparation of outputs for subsequent procurement.

Rather than testing a formal hypothesis, the study uses a longitudinal qualitative case-study design and is organized around the following research question: How are the requirements and institutional conditions of a public innovation procurement process reflected in the development and specification of an AI-enabled socio-technical architecture for industrialized housing delivery? The analysis follows the relationship between institutional requirements, the initial technical response, its formal ex-ante assessment, and subsequent collaborative development, while keeping specification, implementation, testing, and operational validation as distinct evidence states.

The contribution is not the proposal of a new computational technique or a claim that the individual technical components are themselves novel. It is a longitudinal account of how public innovation procurement requirements correspond to the formation and refinement of an AI-enabled socio-technical architecture that connects computational capabilities with data structures, human decision points, governance and interoperability arrangements, and procurement-document preparation. This institutional-to-architectural perspective links research on construction AI and digital integration with innovation procurement and public-sector AI governance at a level that is usually examined separately. The remainder of the paper develops the theoretical basis for this relationship, describes the case-study design and documentary analysis, reports the evolving architecture and its evidential status, and discusses the implications and limits of the pre-deployment evidence.

## 2 Theoretical background

### 2.1 AI-enabled decision support as operational infrastructure

AI can be understood as an operational layer connecting data and models with the rules governing an industrial process. It can combine simulation and optimization with decision procedures (Baduge et al., 2022). This differs from judging AI through the performance of a single algorithm. At system level, the issue is whether individual capabilities work together in a way that supports coordination and repeatable decisions. That question is particularly relevant in sectors where work is distributed among many actors and late changes are expensive, often under strict regulatory constraints. AI can then support earlier coordination, structured comparison, and consistent rule application in such settings (Turner et al., 2021). In practice, its functions usually sit within a wider digital environment that includes BIM and digital-twin technologies (Kor et al., 2023; Sepasgozar et al., 2023). Operational decision support also depends on reliable data and understandable outputs. Interoperability and governance arrangements affect whether users can interpret and act on system-generated information (Omrany et al., 2023; Wang et al., 2024).

What AI can do in practice also depends on its industrial and organizational setting. Regulatory context, data quality, available skills, and institutional capacity all matter (Rashidi et al., 2026). In construction and public delivery, digital transformation combines technical change and standardization with coordination and leadership across organizations (Turner et al., 2021; Gledson et al., 2024). Public-sector AI adds another layer. Its integration affects organizational structures, data practices, work routines, and human-machine relations, making it a socio-technical issue rather than a purely computational one (Tangi et al., 2025). Explainability provides a clear example. An explanation must make sense to the people who receive, interpret, or contest an algorithmic output; technical disclosure alone may be insufficient (de Bruijn et al., 2022). Algorithmic decision-making raises related questions of democratic legitimacy, including how decisions are authorized, scrutinized, and justified (Grimmelikhuijsen and Meijer, 2022).

These concerns widen the criteria used to assess AI-enabled decision support in public settings. Automated administrative decision-making can involve trade-offs among accountability, efficiency, equality, fairness, responsiveness, privacy, rule of law, and transparency (Roehl and Hansen, 2024). Evidence from citizen-facing applications also shows that perceived failures of fairness and transparency can damage evaluations of government, including among people who are not directly affected by the decision (Schiff et al., 2022). Much of this literature concerns automated administrative decisions rather than advisory decision-support systems. The distinction matters. Even so, the same governance questions arise when AI-generated outputs enter public decision processes. Accountability, transparency, context sensitivity, feedback, and related requirements need attention across the system lifecycle, including before deployment (Agbabiaka et al., 2025).

Our focus is the shift from local automation to coordinated decision support across project stages. Outputs need to be traceable and understandable if they are to support administrative or managerial action, and human decision points should remain explicit. An integrated architecture can support earlier requirements checking, information exchange, performance assessment, and multicriteria comparison. These are specified capabilities; they are not evidence of operational performance. Off-site lifecycle architectures have already been used for planning and quality control, maintenance, and logistics (Lee and Lee, 2021). In public industrialized housing, a similar system-level logic can connect design and manufacturing with assessment, procurement, and long-term management. We assess AI here through the integration of computational capabilities with data, workflows, governance arrangements, and human decisions across the delivery process. Architecture-level features alone do not demonstrate trustworthiness or effectiveness. Those claims require operational evidence.

### 2.2 Public innovation procurement and collaborative requirement development

Public innovation procurement is used when public authorities cannot meet a defined need by purchasing an established market solution, or when procurement creates scope for new or substantially improved solutions (Obwegeser and Müller, 2018; Uyarra et al., 2020). Functional procurement makes this distinction more explicit by specifying the problem or function to be addressed rather than prescribing a product. Societal needs and public objectives are translated into functional requirements, while potential suppliers retain scope to determine how those requirements can be met (Edquist and Zabala-Iturriagagoitia, 2020). Innovation does not follow automatically from this approach. Procurement design, market conditions, buyer capabilities, and the degree of specification affect the scope for experimentation, while excessive prescription and limited dialogue can restrict supplier responses (Uyarra et al., 2014; Chiappinelli et al., 2025).

Requirements can change during the procurement process. Problem and solution framings may co-evolve as public buyers, suppliers, and other stakeholders exchange knowledge and reconsider both needs and possible responses (Clarke et al., 2026). Market dialogue can also be used to clarify needs and refine requirements before tendering (Brataas et al., 2022). That interaction does not always produce the same outcome. Different forms of market engagement lead procurers to adapt the subsequent procurement process in different ways as learning takes place (Berkhout et al., 2026). The capabilities needed for this work may be distributed across organizations rather than concentrated within a single procurement unit (Taheriruh et al., 2025). Innovation intermediaries can also connect procurement activity with technical development and institutional change (Selviaridis et al., 2023).

AI procurement introduces requirements beyond conventional technical and commercial specifications. Transparency, accountability, due diligence, institutional capacity, and the allocation of responsibility become part of the procurement problem when public bodies acquire or develop AI systems (Hickok, 2024). Existing procurement practices can affect which AI systems are acquired, who participates in procurement decisions, and how responsibility for AI governance is distributed (Johnson et al., 2025). Contractual requirements can address some of these concerns, but procurement cannot substitute for the wider legal and institutional governance of public-sector AI (Sanchez-Graells, 2024). Organizational, process, technology, and data-management constraints have been identified in the adoption of AI within governmental procurement organizations (Andersson et al., 2025).

Digital procurement in the built environment depends on organizational capability and process adaptation as well as technological availability (Chan and Owusu, 2022). Our argument here is narrower. Procurement requirements do not determine a technical architecture. They define needs, constraints, evaluation criteria, and interaction conditions against which technical alternatives can be specified and revised during collaborative development. On that basis, we examine whether, and in what form, those requirements and institutional conditions are reflected in the functional and computational architecture of an AI-enabled decision-support system before operational deployment.

### 2.3 Industrialized housing delivery as a socio-technical system

Industrialized housing delivery brings together product design, supply-chain management, regulatory compliance, manufacturing, transport, on-site assembly, maintenance, and information management (Li et al., 2019; Hussein et al., 2021). Prefabrication is only one part of this process. Research on modular integrated construction identifies information sharing, early stakeholder involvement, supply-chain-oriented design, and coordination across project stages as recurring requirements for delivery (Wuni and Shen, 2023). Public housing introduces additional constraints related to affordability, regulatory compliance, environmental performance, and accountability. Long-term operation also matters because decisions made during design and production affect subsequent use and maintenance. Repeatable industrialized processes depend on information continuity across the building lifecycle (Lennartsson et al., 2020; Iacovidou et al., 2021). Adoption also depends on policy and market readiness, social conditions, and stakeholder alignment (Gusmao Brissi et al., 2021; Khan et al., 2022).

Recent reviews show that these dependencies remain difficult to manage in practice. Information integration and coordination between project stages remain recurring problems in prefabricated supply chains (Han et al., 2023; Zhang et al., 2024a). A wider review of off-site construction identified 47 barriers to adoption, with lack of skills, weak cooperation across the value chain, project and capital costs, and the absence of common standards among the most frequently reported (Alhawamdeh and Lee, 2025). Research focused specifically on industrialized housing reaches a similar conclusion. Regulatory misalignment, rigid procurement and delivery models, unstable supply-chain capacity, and the absence of standardized components and integrated digital workflows continue to constrain wider uptake (Hedayati et al., 2026). Industrialized housing sits within an organizational and institutional setting that extends beyond factory production.

Digital technologies address parts of this coordination problem, although their use remains uneven across the project lifecycle. Research on digitalized industrialized construction covers design, production, construction, and operation, but adoption differs between stages and the operation phase has received less attention (Fan et al., 2024). BIM-based and other digital approaches can connect design information with production and logistics while supporting performance and sustainability assessment (Cheng et al., 2023). Digital twins extend this logic by linking information across physical and digital processes. In industrialized off-site construction, AI-driven digital twins have been studied for monitoring, scheduling, quality control, logistics, and maintenance, while data integration and interoperability remain recurring implementation issues (Najafzadeh and Yeganeh, 2025). Lifecycle information, reuse potential, and maintenance implications also need to enter project definition before major design choices are fixed (Iacovidou et al., 2021).

Housing raises another issue that is less visible in technology-centered accounts. A review of circular industrialized housing identified governance, financial conditions, site and logistics, construction systems, building information, and cultural factors across the housing lifecycle. The same review found that research is concentrated on design, with less attention to planning, manufacturing, and disassembly, and that affordable and social housing have received limited direct study (Davis et al., 2025). This matters for public housing because technical choices sit within procurement rules, affordability targets, public accountability, and long-term asset management. A decision-support architecture for this setting needs to represent requirements that originate in several parts of the delivery system rather than in design performance alone.

Construction-AI research has largely concentrated on task automation, BIM integration, digital twins, and lifecycle coordination (Baduge et al., 2022; Gill et al., 2024). These approaches provide methods and digital infrastructure for individual parts of industrialized housing delivery, but they do not remove the institutional conditions under which public projects are specified and assessed. Industrialized public housing is a useful setting for examining how procurement and institutional requirements are reflected in the development of an AI-enabled socio-technical architecture. The analysis concerns that pre-deployment relationship. Technical specification and operational performance remain separate forms of evidence.

### 2.4 Positioning of the study within related digital and AI-enabled approaches

Several adjacent research streams address parts of the problem examined here. BIM-enabled workflows, digital twins, AI-based design and optimization, digital industrialized construction, public innovation procurement, and public-sector AI governance differ in their primary unit of analysis and in the extent to which they address institutional and procurement conditions. Table 1 summarizes these differences and positions the present study relative to them.

**Table 1:** Positioning of digital and AI-enabled approaches relevant to industrialized housing delivery.

Approach	Main analytical focus	Typical scope	Institutional/Procurement dimension	Main limitation for the present research question	Representative literature
BIM-enabled workflows	Information modelling, coordination, rule checking and exchange	Design to construction, with increasing lifecycle extension	Usually secondary; procurement may use BIM outputs but is not generally the object of analysis	Does not by itself explain how public requirements become embedded in system architecture	Bakhshi et al. (2022); Cheng et al. (2023); Bayzidi et al. (2025)
Digital twins	Data continuity, monitoring, simulation and feedback between physical and digital assets	Construction and operation, often lifecycle-oriented	Governance and interoperability increasingly considered, but procurement requirements are usually peripheral	Stronger on lifecycle integration than on the pre-deployment translation of institutional requirements	Sacks et al. (2020); Sepasgozar et al. (2023); Alnaser et al. (2024); Karunaratne et al. (2025); Miraj et al. (2025); Najafzadeh and Yeganeh (2025)
AI-enabled design and optimization	Generation, prediction, optimization and comparison of design alternatives	Mainly design and pre-construction	Public-sector requirements normally enter as constraints or criteria rather than as an object of institutional analysis	Often evaluates algorithms or individual functions rather than the architecture linking them to public decision processes	Baduge et al. (2022); Gill et al. (2024)
Digital industrialized / off-site construction	Integration of design, manufacturing, logistics and information flows	Cross-stage project delivery	Organizational and supply-chain conditions are increasingly studied	Persistent fragmentation across organizations, project stages and digital systems	Loo and Wong (2023); Wuni and Shen (2023); Fan et al. (2024); Hedayati et al. (2026)
Public innovation procurement	Public needs, functional requirements, market interaction and development of new solutions	Procurement and innovation process	Central	Strong on requirements, interaction and governance, but generally does not trace these into the functional and computational architecture of an AI-enabled construction system	Edquist and Zabala-Iturriagagoitia (2020); Chiappinelli et al. (2025); Clarke et al. (2026)
Public-sector AI procurement and governance	Accountability, transparency, responsibility, institutional capacity and procurement practice	Acquisition, governance and use of AI	Central	Limited connection between institutional procurement conditions and the detailed architecture of domain-specific AI decision-support systems	Hickok (2024); Sanchez-Graells (2024); Johnson et al. (2025)

Table 1 places the study at the intersection of these streams. We focus on the pre-deployment translation of procurement and institutional requirements into the functional and computational architecture of an AI-enabled decision-support system for industrialized housing. The analysis concerns architectural specification and development rather than operational performance.

## 3 Research design and methods

### 3.1 Study design and unit of analysis

The study uses a longitudinal qualitative case-study design to examine the development of an AI-enabled socio-technical architecture within a public innovation procurement process. Case studies are appropriate when the phenomenon cannot be separated cleanly from its organizational and institutional context and when the purpose is analytical rather than statistical generalization (Yin, 2017). The case is examined before operational deployment. We examine how institutional requirements and procurement conditions are reflected in the specification and development of the architecture over time, rather than the performance of a completed system.

The unit of analysis is the evolving pre-deployment socio-technical architecture collaboratively developed by the consortium. Our focus is its functional and computational configuration, represented by eight interconnected modules and a set of cross-cutting enabling conditions. Procurement requirements, the initial technical proposal, and its formal ex-ante assessment provide the institutional and technical context against which the architecture was subsequently developed and refined. Treating these stages as part of an evolving process allows changes in the architecture to be examined without collapsing evidence produced at different points in time, an issue that is central to longitudinal process research (Langley, 1999). These sources are antecedent inputs to the development process, not evidence of operational effectiveness.

The case was selected purposively because it combines AI-enabled decision support with industrialized housing delivery under public procurement conditions. Environmental requirements, administrative use, and procurement constraints are present within the same development process. Its analytical value lies in the access it provides to relationships that would be difficult to observe if the technical system and its institutional setting were examined separately. Single cases can be especially informative when they provide access to processes and relationships that are otherwise difficult to observe directly (Siggelkow, 2007).

We do not seek statistical generalization. Our claims are confined to the relationships that can be traced within this case and to the evidence available before operational deployment. Operational effectiveness, transferability across procurement settings, and performance after deployment require separate empirical evidence.

### 3.2 Case setting and temporal scope

The case concerns a public innovation procurement process in Spain for the development of an AI-enabled decision-support system for industrialized, publicly supported housing. The contracting authority defined a public delivery problem that combined affordability, industrialization, environmental performance, regulatory compliance, and administrative use. The requested solution was expected to support decisions across several stages of housing delivery rather than operate as a stand-alone analytical tool.

Figure 1 summarizes the decision environment of the case, including the principal stakeholder groups and the institutional, environmental, technical, and delivery conditions within which the architecture was developed. The shaded sectors represent interacting dimensions rather than sequential stages. Citizens are represented as stakeholders in the wider governance environment, but their preferences and perceptions are not part of the empirical evidence examined in this study.

**Figure 1:**
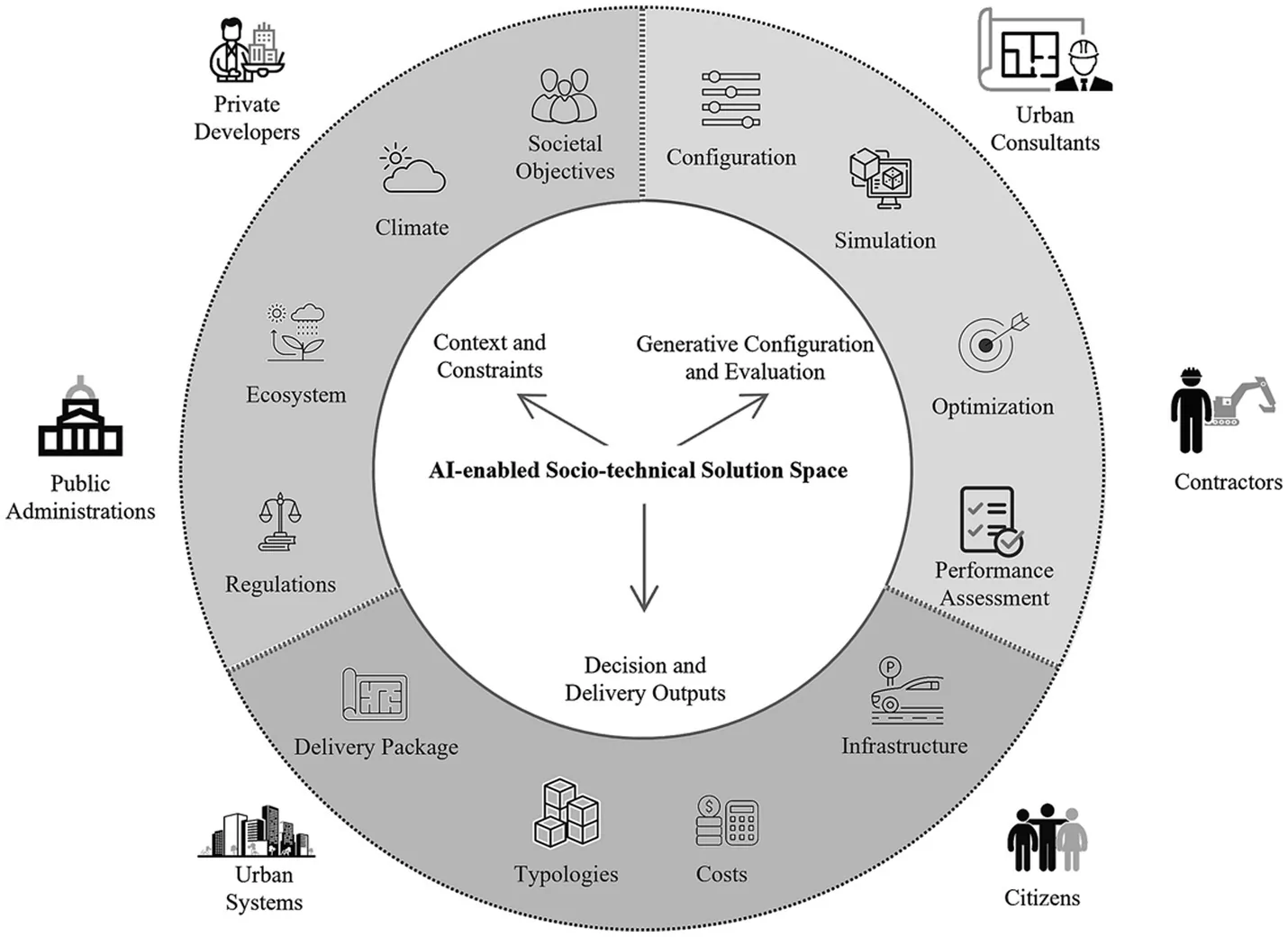
Decision environment of the AI-enabled socio-technical solution space for industrialized housing delivery.

The evidence covers three successive stages of the process. We retain this distinction throughout the analysis because the timing and sequence of evidence matter in longitudinal research and affect how changes and relationships are interpreted (Pettigrew, 1990; Feuls et al., 2023). The first stage covers the initial procurement and proposal process, in which the contracting authority defined the challenge and the consortium formulated its technical response. The second corresponds to the formal ex-ante assessment of that proposal. This assessment records how the proposed solution was evaluated at that point in the procurement process. It is not treated as evidence of technical or operational validation. The third stage covers the subsequent collaborative development of the architecture, during which its functions, workflows, computational arrangements, and cross-cutting enabling conditions were progressively specified and refined. This collaborative development stage took place under a contract awarded following the procurement procedure. The award is treated here only as the institutional condition that enabled the subsequent development work; it is not used as evidence of technical quality, operational effectiveness, or successful adoption.

The stages are retained as separate evidence categories throughout the analysis, with their provenance and temporal position preserved. The empirical corpus covers the development process up to 21 July 2026, which constitutes the study’s documentary cut-off and precedes operational deployment. We do not treat the competitive outcome itself as evidence of scientific or technical performance, and we do not assess the effectiveness of the system in completed housing projects. Implementation, testing, and operational validation are treated as distinct evidence states and are only reported where the available documentation supports them.

### 3.3 Documentary corpus and evidence sources

The empirical material consists of a longitudinal documentary corpus produced by the contracting authority and the consortium at successive stages of the procurement and development process. The institutional subcorpus consists of formally issued records and forms a discrete documentary set. These records establish the public challenge, formal requirements, procedural conditions, and institutional context against which the technical response was developed. By contrast, the collaborative development repository is an evolving working environment containing successive versions and different types of technical material. It is characterized here by evidence category and analytical function rather than by treating every stored file or version as an independent documentary unit.

The corpus comprised 32 discrete documentary records, complemented by a versioned technical working set maintained in the consortium’s shared development environment. The institutional component comprised 12 formally issued records associated with the procurement process. Five contained substantive information used directly to examine institutional requirements and procurement conditions, while seven were used primarily to establish procedural context, provenance, and temporal sequence. The institutional component was supplemented by the consortium’s initial technical proposal and its formal ex-ante assessment. The collaborative-development component comprised 10 meeting records produced between 7 May and 21 July 2026, two detailed planning and task-structure workbooks, one additional planning record, one organizational-structure record, one technical benchmarking record, one monthly development-progress report, and two structured deliverable records from the previous phase. The versioned technical working set comprised successive functional and technical specifications, architecture, workflow and interface documentation, and technical implementation artifacts, including source code where relevant to the architectural functions examined in the study.

The consortium’s initial technical proposal records the first formal technical response to the procurement requirements, including the proposed system concept, functional organization, computational approaches, development strategy, and intended use. The formal ex-ante assessment records the contracting authority’s evaluation of that response at the proposal stage. It is treated as external documentary evidence of formal scrutiny before subsequent development, not as evidence that the proposed functions had been implemented or operationally validated.

The subsequent development material was produced collaboratively and maintained in the consortium’s shared digital workspace. Development was organized through module-specific working groups together with cross-module coordination and management activities. The resulting records document contributions, technical discussions, alternatives, dependencies, and decisions involving multiple consortium partners rather than material produced solely by the authors. Successive working copies were not treated as separate documentary units solely because a new repository version had been created. Where successive versions documented a substantive change relevant to the longitudinal analysis, the change was retained and examined as temporal evidence.

Materials were included when they provided evidence directly relevant to institutional requirements and procurement conditions, the initial architectural specification, formal ex-ante assessment, or the subsequent specification, refinement, or technical implementation of the architecture. Administrative, financial, logistical, and purely organizational communications without direct relevance to these analytical purposes were excluded. Duplicate working copies were excluded. Email correspondence was used to establish provenance, circulation, and temporal sequence where necessary, but was not counted as an independent documentary category.

Meeting records form part of the documentary corpus because they document technical issues, alternatives, agreements, dependencies between modules, and decisions subsequently reflected in the evolving documentation. The meetings themselves are not treated as an independent interview or participant-observation dataset. Functional and technical specifications provide evidence of how individual modules and their interfaces were defined and revised. Architecture and cross-module records provide evidence at system level, including dependencies between modules and arrangements concerning interoperability and governance. Technical implementation artifacts are used only where they help establish the documented development status of a function. Their existence, including the existence of source code, is not treated as evidence of operational performance or validation.

The documentary material was examined using a qualitative document-analysis approach (Bowen, 2009). Documents were differentiated by provenance, temporal position, and evidential function before being compared across development stages. This separation preserves differences in evidential status rather than treating all documentary statements as equivalent evidence. Table 2 summarizes the main evidence categories and their role in the analysis.

**Table 2:** Documentary corpus, coverage and analytical role of the evidence.

Evidence category	Origin	Documentary coverage	Main content	Analytical role	Evidential status
Institutional procurement records	Contracting authority	12 discrete records; 2020–2025	Public need, requirements, procedural conditions, evaluation framework, and institutional context	Establish the institutional requirements and procurement conditions against which the technical response was developed	Institutional requirement
Initial technical proposal	Consortium	1 document; proposal stage	System concept, functional organization, computational approaches, development strategy, and intended use	Establish the consortium’s first formal technical response	Specified in the initial proposal
Formal ex-ante assessment	Contracting authority/evaluation body	1 formal assessment; proposal-assessment stage	Assessment of methodology, planning, technical response, quality, risk, and related proposal dimensions	Document external scrutiny of the proposal before subsequent development	Formally assessed ex ante
Collaborative-development meeting records	Consortium working groups	10 records; 7 May–21 July 2026	Technical issues, alternatives, module decisions, dependencies, coordination issues, and recorded changes	Trace module-level and cross-module development and refinement	Documented development evidence
Development and planning records	Consortium	8 discrete records; development period to 21 July 2026	Task decomposition, work sequencing, module organization, development priorities, benchmarking, progress reporting, and preparation of formal deliverables	Trace how the proposed architecture was decomposed, coordinated, and progressively organized for development	Specified/developed
Functional and technical specifications	Consortium working groups	Versioned technical working set; retained where analytically relevant	Module functions, computational requirements, workflows, interfaces, and technical specifications	Examine how individual functions and interfaces were specified and revised	Specified/developed
Architecture, workflow, and interface documentation	Consortium	Versioned technical working set; retained where analytically relevant	System architecture, module and data relationships, interoperability, workflows, and cross-cutting conditions	Examine system-level integration and architectural refinement	Specified/developed
Technical implementation artifacts	Consortium technical teams	Versioned implementation material, including source code where relevant	Source code and associated implementation material linked to documented system functions	Distinguish functions that entered technical implementation from those remaining at specification or development level	Implemented where directly supported

The corpus comprises 32 discrete documentary records together with a versioned technical working set maintained in the consortium’s shared development environment. Counts refer only to records that could be treated as discrete documentary units. Successive specifications, architecture and interface documents, and implementation artifacts were treated as evolving working objects rather than counted as separate documents solely because a new repository version existed.

### 3.4 Analytical framework and procedure

We organized the analysis around four dimensions already used in the consortium’s work: functional, methodological, institutional, and strategic. These dimensions were not constructed retrospectively as an ex-post coding scheme for the paper. They served as analytical lenses for organizing and comparing evidence across the successive stages previously described. The use of an explicit conceptual framework in qualitative case-study research provides a structure for connecting the research question, empirical material, and its interpretation while retaining the context in which the evidence was produced (Ravitch and Carl, 2019; Gregory, 2020). We used these dimensions to structure the analysis, not as emergent themes or mutually exclusive codes.

The functional dimension concerns the relationships among project inputs, system functions, computational processes, interfaces, and outputs. The methodological dimension concerns how the architecture was specified, developed, reviewed, and progressively refined. The institutional dimension addresses public requirements, administrative use, governance arrangements, and the roles of the organizations involved. The strategic dimension concerns interoperability, procurement readiness, traceability, replicability, scalability, and the conditions associated with wider use. A document or architectural element could contribute to more than one dimension when its implications crossed these analytical boundaries. For analytical clarity, functional elements documented through different project-stage decompositions were consolidated for analysis into eight interconnected modules and three cross-cutting enablers. This representation preserves the documented functions rather than reproducing the numbering used in any single project-stage decomposition.

We applied the framework in four steps. First, the documentary material was separated according to provenance and temporal position: institutional procurement records, the initial technical proposal, the formal ex-ante assessment, and subsequent collaborative development evidence. Preserving this sequence is consistent with process research in which temporal ordering forms part of the analysis rather than being treated as background information (Langley, 1999). Second, the evidence within each stage was examined through the four analytical dimensions. For each document, we considered the origin, purpose, context, and evidential role rather than treating documentary material as a set of equivalent sources (Bowen, 2009). This explicit treatment supports transparency in qualitative document analysis (Wood et al., 2020).

Third, documented public requirements and procurement conditions were compared with elements of the evolving architecture. We looked for identifiable correspondences between an institutional requirement or condition and a functional, computational, governance, or interoperability arrangement documented in the initial proposal or subsequent development material. A correspondence was retained when both sides of the relationship could be located in the documentary record. We do not infer that a procurement condition caused a particular architectural decision. Instead, we examine whether a relationship is documented, where it appears in the development process, and how it is represented in the architecture.

Fourth, statements concerning the system were classified according to their evidential status. Requirements stated by the contracting authority were treated as institutional requirements. Functions described in the initial proposal were treated as specified capabilities at that stage. Statements in the formal assessment were treated as ex-ante evaluation of the proposal rather than as evidence of technical validation. Subsequent specifications, meeting records, architecture documents, and technical artifacts were used to identify later development and, where directly supported, technical implementation. Testing and operational validation were treated as separate evidence states and were not inferred from specification, formal assessment, or the existence of source code.

The analytical dimensions and the questions used to apply them consistently across the corpus are summarized in Table 3. The dimensions overlap where a single architectural decision has implications for more than one part of the system. For example, an interoperability arrangement may have a functional role while affecting governance or future procurement use. Such cases were retained across the relevant dimensions rather than forced into a single category. Interpretations were discussed among the authors and checked against the underlying documentary evidence. We used this cross-checking to verify source provenance, temporal position, and evidential status rather than to claim independent validation. Where documentary evidence differed across stages, the difference was retained and examined as part of the development process rather than reconciled retrospectively. No independent coders were used, and no inter-rater reliability statistics were calculated.

**Table 3:** Analytical dimensions used in the documentary analysis.

Analytical dimension	Main analytical question	Evidence primarily examined
Functional	How are institutional and project requirements represented in system functions, module relationships, computational processes, interfaces, and outputs?	Procurement requirements, initial proposal, module specifications, architecture and interface documentation, technical implementation artifacts
Methodological	How are functions and architectural arrangements specified, developed, reviewed, and refined across successive stages?	Initial proposal, formal ex-ante assessment, development and planning records, meeting records, successive technical specifications
Institutional	How are public requirements, administrative use, governance arrangements, organizational roles, and accountability conditions represented in the architecture and development process?	Procurement records, formal ex-ante assessment, management and cross-module meeting records, governance and workflow documentation
Strategic	What documented arrangements concern interoperability, traceability, procurement readiness, replicability, scalability, and possible extension beyond the immediate development setting?	Procurement documentation, proposal, architecture records, cross-module documentation, development and planning records

To make the requirement-to-architecture comparison traceable, Table 4 summarizes the principal correspondence chains retained in the analysis. For each institutional requirement or condition, the table identifies its documentary stage, the corresponding architectural element, the technical response documented in the case, its development over time, and its evidential status at the documentary cut-off. Only correspondences for which both the institutional and architectural sides could be located in the corpus are reported. Changes over time refer to documented specification, refinement, or analytical consolidation and do not imply that the institutional requirement caused the corresponding architectural decision.

**Table 4:** Traceability matrix from institutional requirements to the evolving architecture.

Institutional requirement or condition	Documentary source/Stage	Corresponding element	Architectural response	Documented development over time	Status at 21 July 2026
Project-, site-, and programme-specific definition of the housing problem	Institutional requirements; procurement stage	M1 Project Definition and Data Input	Structured representation of site, brief, housing programme, targets, constraints, and configuration parameters	Project and data input were retained as a distinct entry point across the proposal and subsequent development decompositions and consolidated analytically as M1	Specified/developed
Dynamic consideration of applicable building, housing, planning, and site constraints	Institutional requirements; procurement stage	M3 Regulatory and Site Context Analysis	Rule-based compliance checking and formalization of regulatory, planning, climatic, and contextual constraints before configuration	A broad requirement for regulatory adaptation was progressively represented as a dedicated context- and compliance-checking function, separated from design generation	Specified/developed; implementation only where directly supported
Open and updateable catalogue of industrialized components and associated product information	Institutional requirements; procurement stage	M2 Data and Component Catalogue; C Interoperability	Structured reusable component data, geometry, attributes, production constraints, and links to external information sources	The catalogue was progressively specified as a reusable information structure supporting configuration and exchange with other system functions	Specified/developed
Generation of industrialized design alternatives from site, programme, catalogue, and regulatory constraints	Institutional requirements; procurement stage	M4 Design Solution Configuration	Generative and parametric configuration of admissible solutions using catalogue elements and formalized constraints	Functions initially represented in broader configuration-and-optimization terms were subsequently differentiated for analysis between design configuration and optimization and scenario evaluation	Specified/developed; selected functions entered implementation
Assessment of energy, carbon, comfort, climatic, and wider environmental performance	Institutional requirements; procurement stage	M5 Environmental and Energy Assessment	Performance assessment integrating energy, thermal comfort, carbon, climatic, and environmental indicators, including surrogate-model-supported estimation where documented	General environmental and simulation requirements were progressively organized as a dedicated assessment function linked to configuration and subsequent optimization	Specified/developed; implementation where directly supported
Assessment of investment, construction, maintenance, and financial feasibility	Institutional requirements; procurement stage	M6 Cost Estimation and Financial Planning	CAPEX, OPEX, maintenance, feasibility, and related financial metrics	Economic requirements were retained as a distinct assessment function and connected to the information entering scenario evaluation and optimization	Specified/developed
Comparison and iterative improvement of alternative solutions across technical, environmental, and economic criteria	Institutional requirements; procurement stage	M7 Optimization and Scenario Evaluation	Evolutionary multi-objective optimization combined with multicriteria scenario comparison and human review of trade-offs	Optimization and scenario-comparison functions documented separately or within broader functional groupings across project stages are represented together analytically, with an iterative feedback connection to design configuration	Specified/developed; selected functions entered implementation
Open standards, BIM-based information management, interoperability, and information continuity across project stages	Institutional requirements; procurement stage	C Interoperability, affecting M1–M8	Open data structures, BIM-related standards, common schemas, interfaces and exchange mechanisms across modules and project stages	Interoperability evolved from a technical requirement and proposal-level connectivity provision into a cross-cutting architectural condition spanning the complete workflow	Specified/developed; interface implementation where directly supported
Use of system outputs in public procurement and preparation of tender documentation	Institutional requirements; procurement stage	M8 Deliverables and Procurement Documentation	Transformation of selected alternatives and associated technical information into interoperable models, reports, spreadsheets, drawings, and procurement-ready outputs	The generation of formal project outputs remained a distinct function across the documented stages and is represented as the endpoint of the analytical workflow	Specified/developed; operational use not validated
Controlled access, accountability, review, approval, and traceability within a public-sector decision environment	Institutional and governance conditions; procurement and development stages	A Security; B Governance	Identity and access control, audit trails, roles, review and approval workflows, accountability, and change control	Functions that appeared as support modules or governance arrangements in proposal and development documents were consolidated analytically as cross-cutting enablers because they apply across the architecture	Specified/developed; operational effectiveness not validated

Documentary source/stage labels are intentionally generic to preserve case anonymity. “Specified” refers to functions documented in the initial technical response or subsequent specifications; “developed” indicates subsequent collaborative refinement supported by development records; “implemented” is used only where technical implementation artifacts support that status. Testing is not inferred from specification, development, or source code, and no system-level operational validation had occurred at the documentary cut-off.

### 3.5 Collaborative development and treatment of discrepancies

The architecture was developed through a distributed collaborative structure rather than by a single technical team. Work was organized through module-specific working groups, together with cross-module technical coordination and management-level coordination when decisions affected broader aspects of the development process. The composition of the working groups varied according to the issue under consideration, and technical contributions were developed jointly by consortium partners. Module-specific meetings addressed the definition and refinement of functions, interfaces, and implementation tasks, while cross-module meetings addressed dependencies and the implications of technical decisions for other parts of the architecture. Shared specifications, meeting records, architecture diagrams, interface definitions, development records, and related technical artifacts were maintained in the consortium’s collaborative workspace as the work progressed. These shared artifacts provided common reference points across specialized groups, consistent with research showing that boundary objects can support knowledge integration across disciplinary and organizational boundaries (Caccamo et al., 2023).

Because the modules were interdependent, decisions made within one technical area could have implications for other parts of the system. Technical alternatives and differences between requirements, proposed solutions, specifications, or successive development decisions were discussed in meetings involving the relevant disciplines and partner organizations. Their implications for connected modules and other technical areas were considered before adopted changes were incorporated into the collaborative documentation. Interorganizational projects can combine event-based, social, technical, and documentary mechanisms to support knowledge sharing across organizational boundaries (He et al., 2023), while differences in professional, technical, and organizational perspectives can produce fragmented understandings that need to be addressed during joint problem and solution development (McCarthy et al., 2021). Knowledge transfer across specialized subteams is relevant to integrative collaboration in complex construction projects (Duva et al., 2024).

The process did not use a formal contradiction register, an independent adjudication protocol, or quantitative confidence scores. Therefore, we do not transform disagreements or alternative proposals into coded events or numerical measures. Instead, meeting records and subsequent technical documentation are used to examine how documented differences were addressed during development. When sources produced at different stages contain different formulations, those differences are retained as temporal evidence of the development process rather than harmonized retrospectively. Consensus, resolution, or influence is not inferred where the documentary record does not support it.

Meeting records and subsequent technical documentation perform complementary roles in this analysis. Meeting records document the issues considered, alternatives discussed, dependencies identified, and decisions recorded at a given stage. Specifications, architecture documents, and technical artifacts provide evidence of the technical state subsequently documented. Recorded decisions and subsequent implementation artifacts can document the development of a function, but they do not by themselves establish its operational effectiveness or validation.

### 3.6 Researcher position, evidence status, and case anonymity

The authors form the scientific research component of the consortium involved in the case and participated directly in the development process. This position provided access to the evolving technical documentation and detailed contextual knowledge of the decisions and interactions surrounding its production. It also carries a risk of interpretive proximity because the researchers are analyzing a process in which they themselves participated. Researcher positionality can affect how qualitative evidence is accessed, interpreted, and represented, making explicit reflection on the researcher’s relationship to the setting part of methodological transparency (Olukotun et al., 2021).

This proximity does not mean that the evidence examined in the study was produced solely by the authors. The technical documentation was developed collaboratively across consortium partners through module-specific working groups, cross-module coordination, and management activities. Institutional procurement documents originated from the contracting authority, and the formal ex-ante assessment constitutes a documentary source external to the consortium’s own technical documentation. We distinguish the provenance of the evidence from the authors’ subsequent interpretation of its implications. Author participation provides contextual understanding of the development process, but claims concerning requirements, architectural functions, development decisions, and implementation status are anchored to the documentary evidence previously described.

The evidential boundaries established throughout the analysis are retained when reporting the case. Institutional requirements, initial specifications, formal ex-ante assessment, subsequent development, technical implementation, testing, and operational validation are treated as distinct states, and evidence from one state is not used to infer another.

The case is reported in anonymized form with the authorization of the contracting organization and participating partners. The name of the procurement initiative, the contracting organization, consortium members, platform identifiers, procurement references, and direct source links are withheld. Some institutional records are publicly accessible, but direct identifiers are not reported in order to preserve the agreed anonymity of the case. The technical and analytical content required to examine the architecture is retained, including its functional organization, computational approaches, development process, evidence categories, and relationships with institutional requirements. Here, anonymization concerns case identification rather than the removal of analytically relevant technical information. Decisions about naming and anonymization in qualitative research involve a balance between protection, representation, and transparency rather than a purely mechanical removal of names (Heaton, 2022).

Case anonymity necessarily limits source-level traceability because individual public records and case-specific technical documents cannot be directly identified in the article. To address this limitation, we report the composition and provenance of the corpus, its temporal structure, the analytical role assigned to each evidence category, and the procedure used to distinguish evidential states. These details allow readers to assess how the interpretations were derived while we respect the agreed reporting conditions. The case-specific collaborative development materials are not publicly available.

## 4 Results: development and specification of the AI-enabled architecture

The results follow the four analytical dimensions defined in the research design. We first describe the functional and computational architecture documented in the case and then examine its development trajectory, institutional and governance arrangements, and provisions for interoperability, procurement use, and possible transfer. Throughout the section, requirements, initial specifications, formal ex-ante assessment, subsequent development, technical implementation, and operational validation are kept analytically distinct.

### 4.1 Functional and computational architecture

The documents describe an architecture that connects project definition with the production of technical and procurement outputs through eight interconnected modules arranged as a sequential decision-support workflow. Figure 2 represents this functional and computational configuration. Project Definition and Data Input (M1) establishes the site, brief, targets, and constraints. Data and Component Catalogue (M2) organizes the modular components, attributes, and geometry available to subsequent modules. Regulatory and Site Context Analysis (M3) introduces applicable regulatory, planning, climatic, and contextual conditions before Design Solution Configuration (M4). Environmental and Energy Assessment (M5) and Cost Estimation and Financial Planning (M6) provide performance and economic information that enters Optimization and Scenario Evaluation (M7). Deliverables and Procurement Documentation (M8) translates the selected configuration and its associated information into technical outputs for subsequent administrative and procurement use.

**Figure 2:**
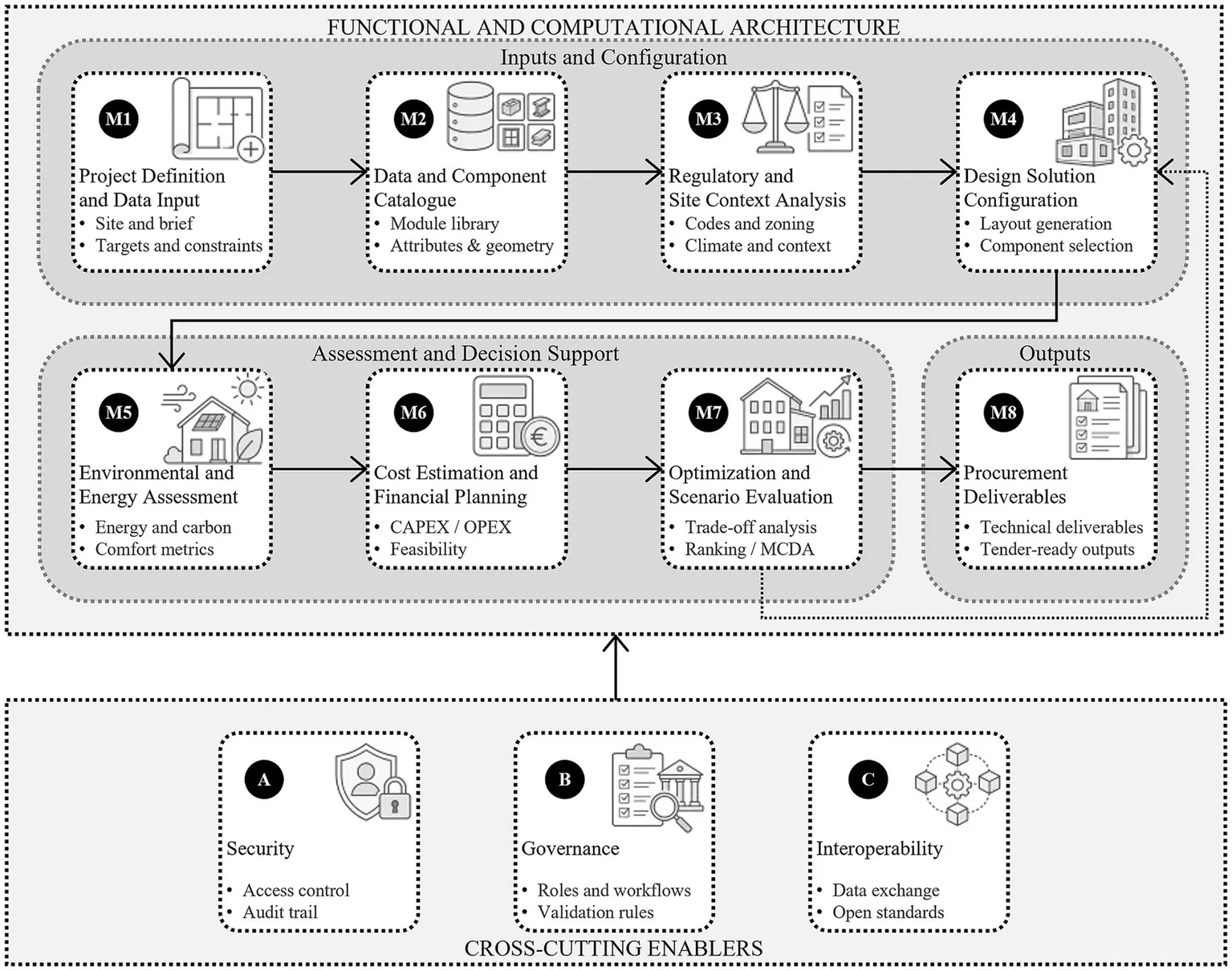
Functional and computational architecture of the AI-enabled decision-support workflow.

The modular architecture is designed to translate heterogeneous project conditions into alternatives that can be compared, traced, and prepared for implementation. In conventional project development, regulatory, environmental, economic, and constructability checks are frequently distributed across separate workflows or undertaken after key design choices have been made (Li et al., 2019; Ammar et al., 2022). The proposal places these checks within a common project environment at an earlier stage. Cognitive digital-twin approaches similarly provide a basis for combining heterogeneous data, lifecycle information, and decision processes within a shared environment (El Mokhtari et al., 2022). Digital twin studies emphasize information continuity from design and construction through operation and maintenance (Ammar et al., 2022; Yang et al., 2024). The framework is designed to compare alternatives while integrating compliance checks and estimates of cost, schedule, energy use, thermal comfort, maintenance implications, and lifecycle environmental performance. Reviews of AI in construction support the early integration of sustainability criteria and multistage evaluation rather than treating them as later compliance exercises (Gill et al., 2024; Ehtsham et al., 2025).

Table 5 summarizes the eight functional modules and the three cross-cutting enablers. The principal rule-based, AI-related and other advanced computational capabilities are concentrated in M3–M5 and M7, covering regulatory and contextual checking, constrained design configuration, performance estimation, multi-objective optimization, and multicriteria scenario evaluation. We do not treat these capabilities as a single AI category. M3 is primarily rule-based, M4 combines rule-based logic with constrained configuration, M5 specifies surrogate-model-supported performance estimation, and M7 combines multi-objective optimization with multicriteria decision support. M1, M2, M6, and M8 provide the project-definition, data-management, economic-planning, and documentation functions required by the workflow. Security, governance, and interoperability operate as enablers across all eight modules. They are not sequential processing stages. Not every module is specified to incorporate AI. Instead, AI-related capabilities are introduced where they support rule application, constrained exploration, performance assessment, optimization, and decision-making. Nor are all advanced computational methods classified here as AI. Table 6 distinguishes the main computational approaches and the human role retained within each of the principal decision-support modules. No formal uncertainty-quantification framework, sensitivity analysis, or Monte Carlo procedure is documented for these functions. Scenario comparison and project-risk management are not treated as substitutes for predictive uncertainty analysis in this study.

**Table 5:** Functional modules and cross-cutting enablers of the framework and their documented functions and outputs.

ID	Element name	Main function	Main outputs or contribution
M1	Project Definition and Data Input	Defines the project brief, site conditions, targets, constraints, and core input parameters.	Structured project brief and configuration data.
M2	Data and Component Catalogue	Organizes modular components, attributes, geometry, and associated technical data.	Structured component catalogue and reusable data library.
M3	Regulatory and Site Context Analysis	Interprets regulatory, planning, climatic, and site-specific conditions that constrain the design process.	Compliance criteria and contextual parameters.
M4	Design Solution Configuration	Generates and configures layouts and component combinations within catalogue and regulatory constraints.	Configured design alternatives.
M5	Environmental and Energy Assessment	Evaluates energy, carbon, thermal comfort, climatic, and environmental performance.	Performance indicators and assessment results.
M6	Cost Estimation and Financial Planning	Estimates CAPEX, OPEX, project feasibility, and wider financial implications.	Cost plans and financial metrics.
M7	Optimization and Scenario Evaluation	Combines multi-objective optimization with multicriteria comparison across technical, environmental, economic, and public-value criteria.	Ranked scenarios, trade-off analysis, and decision-support metrics.
M8	Deliverables and Procurement Documentation	Translates selected alternatives into interoperable technical outputs and tender-ready documentation.	BIM models, CAD drawings, spreadsheets, technical reports, and procurement packages.
A	Security	Controls identity, access permissions, audit trails, and the protection of project information.	Secure access and traceable user actions.
B	Governance	Defines roles, workflows, review and approval rules, accountability, and change-control arrangements.	Traceable decisions and controlled review and approval workflows.
C	Interoperability	Supports data exchange, open standards, interfaces, and information continuity across tools and project stages.	Interoperable information and reusable data flows.

**Table 6:** AI-related and advanced computational capabilities, documented computational approaches, method classes, and human roles in the main decision-support modules.

ID	Computational capability	Computational approach	Method class	Human role
M3	Regulatory and contextual checking	Rule-based compliance checking, contextual data integration, and formalization of regulatory constraints.	Rule-based inference and contextual data integration	Reviews applicable requirements and interpretations and resolves exceptions.
M4	Constrained design configuration	Generative and parametric configuration over the modular component catalogue under formalized constraints, with evolutionary operators for generating and modifying admissible configurations.	Generative / algorithmic configuration with evolutionary search	Defines design requirements, reviews generated alternatives, and selects viable configurations.
M5	Performance estimation	Pre-trained surrogate models using machine- and deep-learning techniques for rapid prediction of energy, comfort, and other selected performance indicators, complemented by detailed simulation where required.	Machine-learning/deep-learning surrogate modelling and simulation	Reviews assumptions, input conditions, and assessment results.
M7	Optimization and scenario evaluation	Evolutionary multi-objective optimization using genetic algorithms, including NSGA-II as specified in the technical documentation, combined with multicriteria decision-support methods.	Evolutionary optimization and multicriteria decision support	Sets objectives and criteria weights, reviews trade-offs, and makes the final selection.

M1, M2, M6, and M8 provide essential project-definition, data-management, economic-planning, and documentation functions, while A–C operate across the complete architecture as cross-cutting enablers. The classification distinguishes rule-based, machine-learning, evolutionary-optimization, and multicriteria methods rather than treating all advanced computational functions as AI, reporting documented computational approaches, not evidence of operational validation.

The functional decision-support core combines rule-based regulatory checking, constrained configuration, surrogate-model-supported performance estimation, cost and financial analysis, optimization, and multicriteria evaluation within a human-in-the-loop process. Its eight modules form an iterative sequence. Project definition and data input (M1) establishes the brief, while the Data and Component Catalogue (M2) provides reusable product information. Regulatory and Site Context Analysis (M3) constrains Design Solution Configuration (M4). Environmental and Energy Assessment (M5) and Cost Estimation and Financial Planning (M6) feed Optimization and Scenario Evaluation (M7), after which the selected alternative is translated into Deliverables and Procurement Documentation (M8). A feedback connection from M7 to M4 provides iterative refinement, allowing the design configuration to be revised in response to scenario evaluation before an alternative is selected.

Within this sequence, the standardized catalogue and the rule-based configuration environment play complementary roles. The catalogue provides the solution space with industrial and constructional coherence, while the configuration environment applies this product logic to site-specific and regulatory conditions. In industrialized housebuilding, standardized components become more useful when lifecycle information is managed through shared digital structures (Lennartsson et al., 2020). Higher BIM maturity has also been associated with improved modularization, standardization, data management, and process consistency (Bayzidi et al., 2025). The system is not intended merely to produce design alternatives in isolation. It also coordinates, at specification level, the rules and components used to generate each solution with the indicators and outputs used to assess it. Users can then compare alternatives before committing to a project route. Human judgement remains responsible for selecting objectives, reviewing results, and resolving trade-offs. This emphasis on human judgement is consistent with studies of human-centered digital twins and explainable AI that treat user interaction and operational usefulness as components of system performance (Bäcklund et al., 2024; Kim et al., 2024). Figure 3 situates the functional and computational architecture within public decision-making, procurement, implementation, and organizational learning.

**Figure 3:**
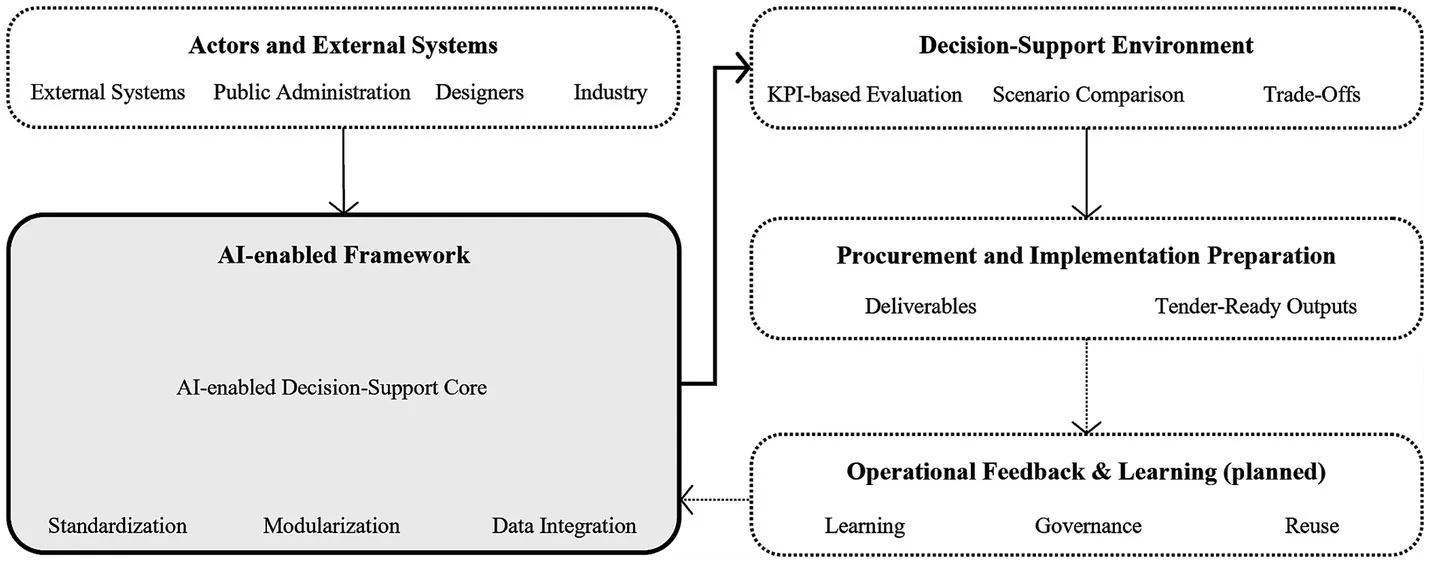
Socio-technical positioning of the AI-enabled framework across decision support, procurement, implementation preparation, and planned operational feedback.

### 4.2 Progressive development and validation logic

The development process was specified in the initial technical proposal as three stages: proof of concept, prototype development, and demonstration and deployment. This sequence was intended to allow requirements, technical functions, and institutional use to be reviewed as the system progresses. Digital twin adoption is commonly organized as a staged process, with validation and organizational preparedness developing alongside the technology (Alnaser et al., 2024; Wang et al., 2024). Figure 4 summarizes the documented development sequence and its planned feedback loops. The stages were specified to be connected through alpha and beta testing, KPI review, and successive revisions across the functional core, particularly the component catalogue, regulatory constraints, configuration environment, and assessment models. The process is iterative by design: technical refinement and institutional validation are intended to proceed together, and deployment readiness was to be assessed through successive tests. Figure 4 also represents the documented development and validation roadmap rather than evidence that all stages had been completed at the empirical cut-off.

**Figure 4:**
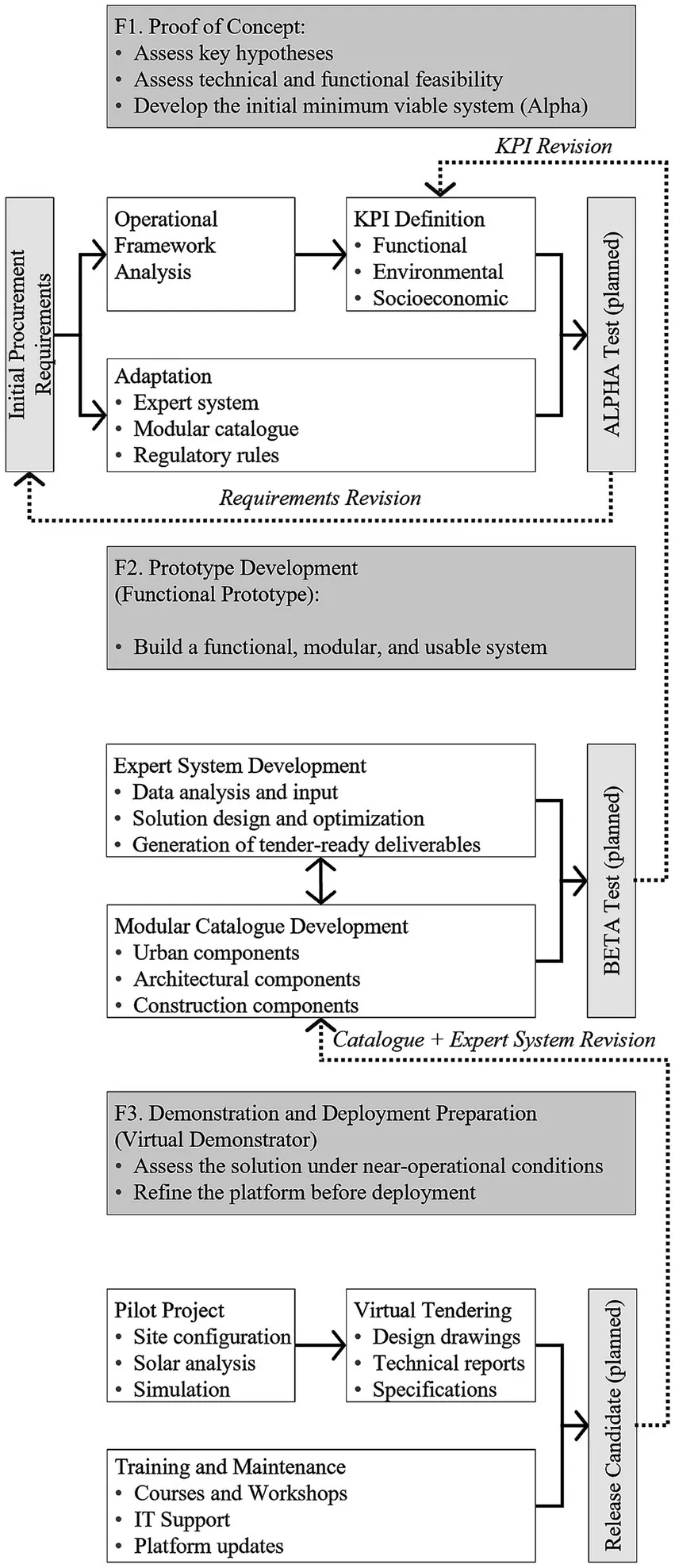
Documented staged development and validation roadmap of the AI-enabled framework.

Phase 1 was specified to focus on defining the operational problem and testing the initial assumptions. Workshops with the public authority were included to establish the scope, functional requirements, and KPI families. An alpha test was planned to identify omissions and revise the initial configuration. The planned role of this phase was to translate a broad public challenge into requirements that can be represented within the framework and tested during subsequent stages.

Phase 2 was specified as the stage for developing a functional prototype by connecting the modular catalogue with the configuration and decision-support environment. Backlogs, iterative prioritization, and recurring demonstrations were specified to support technical development and review. The prototype was to be tested against public requirements and sector expectations in a controlled setting and to introduce a structured comparison of functional, environmental, and socioeconomic indicators.

Phase 3 is planned to apply the system to a specific public site and prepare a pilot project for procurement. The work includes simulation, the generation of documentation for virtual tendering, training, technical support, and preparation of a release candidate. Under the documented development plan, this stage would extend assessment from internal functionality toward use in a public-sector setting.

Subsequent collaborative records show that the architecture did not remain solely at the level of the initial proposal. Module-specific and cross-module working groups progressively refined functions, interfaces, workflows, and system-level arrangements, while technical implementation artifacts provide evidence that some functions entered implementation. The available corpus, however, does not support treating every specified function as implemented or tested, nor does it establish system-level operational validation. Development status is assigned from the evidence available for each function rather than inferred from the planned Alpha, Beta, or demonstration stages.

Successive project records do not reproduce a single fixed module decomposition. The initial proposal and the subsequent development records group some functions differently, particularly project and data input, identity and security, design configuration and optimization, scenario comparison, and cross-cutting governance and interoperability. Despite these differences in decomposition, the underlying functional sequence remains traceable across the corpus. The eight-module architecture and three cross-cutting enablers reported here consolidate these documented functions for analytical comparison rather than representing a formally recorded change from one fixed module count to another.

The documented development approach treats implementation as both a learning process and a software-development problem. The process was designed to consider technical refinement and organizational uptake together, with user participation, validation, and training linking both. Usability, stakeholder engagement, and the establishment of new routines influence long-term impact (Miraj et al., 2025). Table 7 summarizes how the development stages and evidence available at the empirical cut-off are interpreted in this study.

**Table 7:** Development and evidential status of the architecture at the empirical cut-off.

Evidence state	Evidence available in the case	Status assigned in the analysis
Institutional requirements	Procurement specifications and associated institutional records	Required
Initial technical response	Initial technical proposal, including system concept, computational approaches, and phased development plan	Specified in the initial proposal
Formal proposal assessment	External assessment of methodology, planning, technical response, quality, and risk	Formally assessed ex ante
Collaborative architecture development	Meeting records, functional and technical specifications, architecture, workflow, interface, and development documentation	Specified/developed according to the documentary record
Technical implementation	Source code and other implementation artifacts where available for particular functions	Implemented only where directly supported by technical artifacts
Testing	Evidence considered only where an executed test is documented; planned Alpha, Beta, or demonstration activities are not treated as completed tests	Tested only where directly documented
Operational validation	No completed system-level deployment evidence within the empirical cut-off	Not operationally validated

### 4.3 Interoperability, traceability, and procurement readiness

The passage from system outputs to procurement and delivery depends on interoperability, traceability, and procurement readiness. Interoperability, represented as cross-cutting enabler C in Figure 2, applies across all eight functional modules because the framework is intended to produce information for procurement and delivery, not only for design comparison. In industrialized housing, outputs are intended to remain usable as a project moves from early definition to manufacturing, administrative review, contracting, and construction. Effective BIM–prefabrication workflows require compatible systems and coordinated project objects (Li et al., 2019). Within the documented architecture, interoperability includes software exchange but is not limited to it. A common information structure is specified to preserve the links between modular components and design decisions. It also connects, at specification level, regulatory and environmental results with cost data and formal documentation. The specified structure links configuration, checking, comparison, and output generation. It can also make explicit the source of a decision and the assumptions under which an alternative was assessed. Traceability is distributed across the three cross-cutting enablers: security specifies access control and audit trails; governance defines roles, review and approval rules, accountability, and change control; and interoperability preserves information continuity across tools and project stages.

Fragmented data and organizational silos remain common barriers to integrated decision-making in the built environment (Berlato et al., 2025; Chatsuwan et al., 2025). The intended workflow incorporates procurement readiness as part of this response. In conventional practice, fragmented information frequently requires reorganization or reformatting before it can support tendering, administrative review, or construction (Chatsuwan et al., 2025). The proposal structures alternatives so that technical, environmental, and economic assessments can be carried forward into administrative documents and interoperable deliverables. Project information is specified to retain design content, regulatory consistency, industrial relevance, and contractual usability. The technical proposal treats transparency, explainability, and human control as governance and decision-support requirements. The architecture is specified so that officials and technical users can examine how alternatives were generated, the constraints applied, and the trade-offs represented by their performance indicators. Final selection remains with the professional user rather than the computational system. Human-centered evaluations of explainable AI judge explanations by their contribution to interaction and task performance in a defined context (Kim et al., 2024).

Within the documented architecture, procurement readiness depends on information remaining reviewable, comparable, and transferable as it passes from design configuration and assessment to administrative and procurement outputs. M8 provides the endpoint of this sequence by translating selected alternatives and their associated information into interoperable technical deliverables and tender-ready documentation. The procurement documentation specifically requires compatibility with open standards and the extraction of project information for use in public tender documentation. These arrangements are documented as requirements and architectural provisions; their effectiveness in completed procurement and delivery processes is not evaluated within the empirical cut-off.

### 4.4 Security, governance, transfer, and conditions for scalability

Security and governance are represented in Figure 2 as cross-cutting enablers A and B because they apply across the entire functional core. Security covers identity and access control, protection of project information, and audit trails. Governance defines roles and workflows, together with the rules used for review and approval, accountability, and change control. Both are integral to documented architecture and development approach. The proposal specifies iterative interaction with the public authority, review with intended users and sector actors, and training as mechanisms for supporting institutional use. Sustained adoption of digital twins in the built environment appears to hinge on governance, user perspectives, and coordination across organizations (Bäcklund et al., 2024; Miraj et al., 2025). It also provides for manuals and user guidance, together with technical assistance and longer-term maintenance. Future updates are also anticipated. The planned transfer arrangements include user training, technical support, maintenance guidance, and access to the source-code repository so that the contracting organization can continue to maintain and modify the system after the development period. These provisions concern the organizational conditions associated with transferring the system from consortium-led development to subsequent institutional use. Standards, training, technical support, and organizational capability have also been identified as important components of digital twin readiness and long-term implementation (Alnaser et al., 2024; Karunaratne et al., 2025). The evidence documents these provisions for continued use, but does not establish their effectiveness after operational deployment.

The intended scope extends beyond a single pilot. The architecture, catalogue, and development process are specified with future reuse across housing projects and, potentially, other public-sector settings in mind. Replication will still require adaptation to local regulations, data, procurement practices, and organizational capacity; it cannot be inferred from technical functionality alone. At an industrial level, scalability concerns the possible reuse of product logic, interfaces, review procedures, and decision records across projects. Industrialized housebuilding requires continuity across interfaces and feedback throughout the lifecycle to achieve repeatability (Lennartsson et al., 2020). In the case documents, scalability is linked to planned arrangements for governance, maintenance, training, and organizational learning. The technical proposal also includes a post-project roadmap, continued maintenance and updating, pilot-based extension, and provisions for adaptation to other institutional contexts. These elements document a pathway for possible transfer and scaling rather than evidence that either has already occurred. Whether this potential is realized must be tested through repeated deployment and independent evaluation.

## 5 Discussion

The results indicate how procurement and institutional requirements are represented in the functional, computational, and governance arrangements of the evolving architecture. We discuss these correspondences in relation to construction AI, digital integration, public innovation procurement, and public-sector governance. Because the evidence precedes operational deployment, our interpretation concerns the significance of the documented architecture and development process rather than measured effects on project or organizational performance.

### 5.1 From design automation to industrial operating logic

Much of the AI literature in construction has focused on defined tasks such as rule checking, performance simulation, scenario generation, and geometric optimization (Baduge et al., 2022; Gill et al., 2024). Within the case, the documented architecture combines rule-based regulatory and contextual checking with generative and parametric design configuration, surrogate-model-supported performance estimation, cost and financial analysis, evolutionary multi-objective optimization, and multicriteria scenario comparison. As Table 5 shows, these functions involve different computational classes rather than a single category of AI. Together, these functions bring regulatory and economic information into a configuration environment based on modular components and open standards. The same setting supports energy and carbon assessment, together with comfort and other project-performance criteria. These functions explain what the system is specified to calculate or compare, but they do not by themselves explain its industrial significance.

What matters analytically is how the functions are connected. The workflow begins with project definition and a reusable component catalogue. Regulatory and site-context analysis then constrains design configuration, which is followed by environmental, energy, and economic assessment. Optimization and scenario evaluation integrate these results before the selected alternative is translated into deliverables and procurement documentation. The three cross-cutting enablers support the complete sequence. Digital twin research describes a comparable shift when data and decisions are connected across project stages instead of being used in separate analytical tasks (Sacks et al., 2020). We interpret this configuration as an industrial operating logic for preparing, comparing, and documenting project alternatives. Its significance lies in the relationships among the functions and their connection to subsequent administrative and procurement activities, rather than in the performance of any individual computational component.

As discussed earlier in the theoretical background, the case also differs from adjacent digital approaches in the way institutional procurement requirements are connected to the architecture. BIM-enabled workflows provide information structures for modelling, coordination, checking, and exchange, while digital twins extend information continuity toward physical–digital interaction and lifecycle use (Sacks et al., 2020; Cheng et al., 2023; Sepasgozar et al., 2023). AI-based design and optimization research more often examines generation, prediction, or optimization as defined computational tasks (Baduge et al., 2022; Gill et al., 2024). The architecture examined here combines elements from these streams within a pre-deployment decision process in which regulatory constraints, modular product logic, performance and economic assessment, scenario comparison, governance, and procurement outputs are connected. The distinction is architectural and socio-technical rather than a claim that the individual computational methods are themselves novel.

Here, AI-enabled industrial transformation refers to a wider arrangement in which project knowledge can be structured, alternatives generated and assessed, and selected configurations translated into project and procurement outputs. The emphasis is on the organization of decision processes around connected computational, data, governance, and human components. Within that arrangement, AI-related capabilities help organize project knowledge so that alternatives can be generated, checked, compared, and translated into project and procurement actions. Figure 3 represents this mediating role by connecting the technical framework with actors and external systems, the decision-support environment, procurement and implementation preparation, and planned operational feedback. Comparable system-level roles have been described for AI-driven digital twins in industrialized off-site construction, where lifecycle coordination, logistics, interoperability, and decision support are treated as principal system-level contributions (Najafzadeh and Yeganeh, 2025). The evidence from the present case concerns the architecture and documented development and intended use of this operating logic. Performance at scale remains to be demonstrated.

### 5.2 Public innovation procurement as an institutional setting for AI-enabled development

The procurement process provides the institutional setting within which the architecture is specified and developed. The public brief requires support for the design, execution, and maintenance of modular, industrialized, publicly supported housing with nearly zero-energy building performance, together with the assessment of cost, time, energy use, thermal comfort, carbon, and emissions. This creates a challenge spanning technical and industrial development, environmental performance, and administrative use. Public procurement can act as a demand-side innovation instrument in this way, creating scope for the development of solutions that are not available as established products (Obwegeser and Müller, 2018; Uyarra et al., 2020). In the case analyzed, the functional requirements correspond to architectural arrangements for integrating construction, environmental, and economic data, incorporating climatic and regulatory constraints, and producing outputs relevant to fabrication, assembly, supply coordination, and tendering. The correspondence is visible across several parts of the architecture: regulatory and site requirements enter M3 and M4, performance and economic criteria are represented in M5–M7, and procurement requirements are carried into M8 and the cross-cutting interoperability and governance arrangements. Public organizations have been shown to influence innovation precisely through such functional requirements, structured interaction, and arrangements for adoption (Selviaridis et al., 2023). The present case does not establish that the procurement conditions caused particular architectural choices. Instead, institutional requirements and architectural arrangements can be traced on both sides of the development process.

The relationship developed over time rather than through a single translation of the initial brief. Functional procurement leaves suppliers scope to formulate technical responses to a defined public problem (Edquist and Zabala-Iturriagagoitia, 2020), while problem and solution framings may evolve as buyers and suppliers exchange knowledge during innovation procurement (Clarke et al., 2026). The same sequence is visible in the case: the initial proposal was followed by collaborative development in which module-specific and cross-module working groups refined functions, interfaces, workflows, and governance arrangements. The resulting architecture is better understood as an evolving technical response to an institutional problem definition than as a direct implementation of a fixed specification.

The formal ex-ante assessment adds a separate institutional perspective. The assessment considered methodological quality, sustainability, innovation, work planning, and technical detail rather than computational novelty alone. Its evidential role is limited to showing how the proposed response was assessed before subsequent development. It does not demonstrate that the functions were technically validated or that the resulting architecture was effective in use. The breadth of the assessment indicates that the procurement process evaluated the proposed system as more than a collection of algorithms. Methodological feasibility, implementation planning, sustainability, and institutional applicability formed part of the assessment context. Beyond this case, organizational preparation for public-sector AI extends beyond technical capability and includes process, technology, and data-management conditions (Andersson et al., 2025).

### 5.3 Public-value criteria beyond initial cost: affordability, industrialization, energy, and carbon in a combined decision framework

The case shows how several institutionally defined public-value criteria are represented within the decision architecture. Each project alternative can be examined from economic and environmental perspectives while also accounting for thermal comfort, industrial feasibility, and anticipated operational implications. The framework combines functional, environmental, socioeconomic, and cost indicators when comparing alternatives, with CAPEX and OPEX estimates, KPIs, financial plans, and procurement outputs considered alongside geometry and design configuration. Within this structure, alternatives can be discussed in terms of cost, energy use, carbon, thermal comfort, industrial feasibility, and longer-term operational implications within the same decision process. Digital evaluation can link environmental, social, and economic performance when the assessment framework is designed across these dimensions, as research on Construction 4.0 and digital twins has shown (Balasubramanian et al., 2024; Zahedi et al., 2024). The architecture provides this comparison through rule-based checking, surrogate-model-supported performance estimation, economic assessment, KPI-based scenario evaluation, and multi-objective optimization. These computational functions structure the information presented to decision-makers, while the weighting of criteria, interpretation of trade-offs, and final selection remain with professional and administrative users. At this stage, the contribution lies in representing heterogeneous criteria within a common decision process, rather than evidence that the resulting choices improve social, environmental, or economic outcomes.

The architecture makes competing institutional objectives available for explicit comparison within a documented workflow. Such a framework can make trade-offs among affordability, environmental performance, industrial feasibility, and long-term operation visible to decision-makers. Related work has linked digital twins to wellbeing, resilience, inclusion, and long-term performance through broader public outcomes (Omrany et al., 2024). Here, these wider outcomes are not measured. The evidence shows how corresponding criteria and objectives are represented in the architecture and made available for comparison before deployment.

That boundary matters particularly in a public-sector setting. Public value cannot be inferred solely from the presence of criteria labelled as social, environmental, or economic. AI-supported public decision-making can involve tensions among efficiency, accountability, fairness, equality, transparency, privacy, and responsiveness (Roehl and Hansen, 2024), while the legitimacy of algorithmic decision processes also depends on how decisions can be scrutinized and justified (Grimmelikhuijsen and Meijer, 2022). We examine how institutionally specified objectives enter the architecture. We do not assess whether citizens or future residents regard the resulting trade-offs as legitimate, fair, or desirable. Citizens are represented in the wider decision environment shown in Figure 1, but their perceptions and preferences are outside the empirical evidence analyzed here. Direct assessment of those perspectives would require a separate post-deployment or participatory study.

### 5.4 Conditions for institutional scalability and long-term use in public housing systems

Technical performance alone does not determine whether the framework can be maintained, transferred, and reused as technical and institutional requirements evolve. The proposal addresses this requirement through governance and maintenance arrangements, version control, training, technical roadmaps, pilot-site planning, financial planning, and wider partnership development. These provisions shift the question from whether a prototype functions once to whether an organization has the arrangements required to use, maintain, update, and potentially reuse it across successive projects. We use institutional scalability here to describe this potential organizational capacity: preserving technical knowledge, supporting users, adapting the framework, and incorporating it into public-sector routines across successive projects. The case documents provisions directed toward these conditions; it does not demonstrate that such scalability has been achieved. Organizational studies of digital twins identify governance, accessibility, user perspectives, and integration into routine practice as underdeveloped but important aspects of deployment (Miraj et al., 2025).

Interoperability, regulatory alignment, and long-term asset management place AI-enabled decision support within a wider digital and organizational infrastructure. What matters here is less the presence of individual AI functions than whether information, decisions, and responsibilities can remain connected across tools, organizations, and project stages. AI-enabled digital twins provide a useful comparison: their system-level role has been associated with information continuity, governance, interoperability, and lifecycle coordination rather than with isolated computational functions alone (Tavakoli et al., 2024). Digital readiness studies place organizational capacity alongside the technical infrastructure required for implementation. Management support, suitable policies, and professional skills remain important throughout the project (Alnaser et al., 2024; Naji et al., 2024). For public housing, these conditions require coordination among authorities, technical operators, designers, contractors, manufacturers, information managers, and support services. The case also marks a limit to architecture-level claims about AI-enabled industrial transformation. More capable computational models do not by themselves establish durable organizational change. Continued use would depend on institutional resources, maintenance responsibilities, technical support, data and interoperability arrangements, professional capabilities, and coordination across the wider delivery system (Loo and Wong, 2023; Soltani et al., 2025). The planned post-project roadmap, training, maintenance, source-code transfer, and provisions for adaptation to other contexts provide evidence that these issues were considered during development. Whether they support repeated use, transfer across organizations, or wider industrial effects remains an empirical question for subsequent deployment and comparative evaluation.

## 6 Conclusion

This study examines how AI-related and other advanced computational capabilities are incorporated into an evolving socio-technical architecture for industrialized public housing delivery within a public innovation procurement process. The documented architecture combines an eight-module functional decision-support core with three cross-cutting enablers: security, governance, and interoperability. The core links project definition to procurement documentation through iterative configuration, performance and economic assessment, and scenario evaluation. Its significance lies less in the automation of individual tasks than in coordinating these functions within a traceable workflow and specifying how project alternatives can be assessed and carried forward into technical and procurement outputs. These arrangements support our interpretation of AI as part of an industrial operating logic. The evidence concerns a framework under development and does not establish system-level operational effectiveness or performance at scale.

Public innovation procurement is central to the case. The contracting authority defined a challenge in which industrialization, energy performance, carbon reduction, traceability, and future deployment were required to be addressed together. We identify correspondences between these institutional requirements and the functional, computational, governance, and interoperability arrangements subsequently specified and developed within the architecture. The case indicates that the technical architecture cannot be interpreted independently from the institutional setting in which requirements, evaluation criteria, review arrangements, and intended forms of use were defined. We interpret this as an architectural and developmental relationship rather than evidence that procurement conditions caused particular technical choices or produced operational outcomes.

The framework incorporates institutionally defined public-value criteria through a combined set of technical, environmental, economic, and administrative criteria. Industrialization, energy performance, carbon, thermal comfort, cost, and replicability are considered within a shared decision process. This combination is particularly relevant to public housing, where affordability must be balanced with accountability, regulatory compliance, and long-term operation. Within the documented architecture, computational functions structure information and make trade-offs available for review, while final choices remain with professional and administrative decision-makers. These arrangements do not demonstrate that broader public outcomes have been achieved or that citizens and future residents regard the resulting trade-offs as fair, legitimate, or desirable. Such perceptions were not part of the empirical evidence examined in this study.

The study has several limitations. It examines a single purposively selected case, relies primarily on documentary evidence produced within the procurement and development process, and ends before system-level operational deployment. Because the design does not include a failed or comparison case, it cannot establish whether the documented architecture is superior to alternative approaches or predictive of successful adoption. The authors were involved in the scientific component of the consortium, which provides close access to the development process but requires the evidential boundaries and provenance of the documentary material to remain explicit. Future research should test the framework across multiple projects and institutional settings and assess its effects on cost, time, environmental performance, decision quality, procurement processes, organizational learning, and maintenance. Further work should examine implementation fidelity, user interaction, explainability in practice, organizational adoption, and the perceptions of citizens or residents where public-value claims are made. Longitudinal and comparative evaluation will be necessary before claims of replicability, institutional scalability, or long-term industrial transformation can be confirmed. Independent post-deployment evaluation is needed to assess whether the specified governance, interoperability, optimization, and decision-support arrangements perform as intended in practice. The case provides a longitudinal account of how procurement and institutional requirements correspond to the development and specification of an AI-enabled socio-technical architecture for industrialized housing delivery. Its contribution is to make those relationships, architectural arrangements, and evidence states explicit before operational deployment.

## Data Availability

The original contributions presented in the study are included in the article/supplementary material, further inquiries can be directed to the corresponding author.
